# The photoplethysmographic amplitude to pulse pressure ratio can track sudden changes in vascular compliance and resistance during liver graft reperfusion: A beat-to-beat analysis

**DOI:** 10.1097/MD.0000000000007045

**Published:** 2017-06-02

**Authors:** Wook-Jong Kim, Jung-Won Kim, Young-Jin Moon, Sung-Hoon Kim, Gyu-Sam Hwang, Won-Jung Shin

**Affiliations:** a Department of Anesthesiology and Pain Medicine, Laboratory for Cardiovascular Dynamics and Signal Processing, Asan Medical Center, University of Ulsan College of Medicine, Seoul; b Department of Anesthesiology and Pain Medicine, Catholic Kwandong University International St. Mary's Hospital, Incheon, Korea.

**Keywords:** beat-to-beat analysis, liver transplanation, photoplethysmography amplitude to pulse pressure ratio, vascular compliance and resistance

## Abstract

During liver transplantation, the thermodilution cardiac output (CO) technique cannot respond to sudden hemodynamic changes associated with postreperfusion syndrome. Photoplethysmography (PPG) can reflect changes in intravascular volume and thus can be used to assess vasomotor tone and arterial stiffness on the pressure–volume relation. We investigated whether a beat-to-beat analysis of the arterial pressure–PPG relationship can estimate dynamic changes in vascular characteristics immediately after liver graft reperfusion.

In 10 recipients, arterial blood pressure and PPG waveforms recorded simultaneously were analyzed from the beginning of fall to nadir in systolic blood pressure immediately after reperfusion. On a beat-to-beat basis, we compared the ratio of the amplitude of PPG to arterial pulse pressure (PPG_amp_/PP, as relative vascular compliance) to total peripheral resistance (TPR) and Windkessel compliance (C_wk_) obtained from the Modelflow CO algorithm.

Following graft reperfusion, PPG_amp_/PP and C_wk_ increased (median 41.5%; *P* = .005 and 42.0%; *P* < .001, respectively), whereas TPR decreased (median −46.4%; *P* < .001). Beat-to-beat PPG_amp_/PP was negatively correlated with TPR (median *r* = –0.80 [95% CI –0.85 to –0.76] on linear regression and *r*^2^ = 0.84 [95% CI 0.73–0.92] on curvilinear regression), and was positively correlated with C_wk_ (median *r* = 0.86 [95% CI 0.81–0.91] on linear regression and *r*^2^ = 0.88 [95% CI 0.75–0.96] on curvilinear regression).

Our results suggest that relative compliance, obtained from beat-to-beat analysis of PPG and arterial pressure waveforms, can track abrupt changes in vascular characteristics associated with postreperfusion syndrome. This simple index would contribute to differential diagnoses of sudden hypotension.

## 1 Introduction

When hypotension develops suddenly during an operation, it is helpful to assess the compliance of the arterial system to decide which treatment is appropriate for the patient (e.g., fluid, inotropic, or vasoactive drugs). Particularly during liver transplantation, hemodynamic deterioration caused by factors such as postreperfusion syndrome is frequently observed in up to 50% of patients.^[1,2]^ Postreperfusion syndrome is comprised of severe hypotension, bradycardia, and rapid changes in cardiac output and/or systemic vascular resistance even a few minutes.^[3]^ Restoration of blood pressure and systemic vascular resistance with vasoactive drugs ensures adequate perfusion to the liver graft and vital end organs; however, the excessive use of vasoconstrictors may lead to severe ischemic complications such as renal failure.^[4,5]^ Therefore, accurate and timely assessment of arterial compliance and resistance is of interest to optimize vascular tone for adequate perfusion pressure.

Although various less invasive hemodynamic monitoring devices that employ arterial pressure waveform analyses have been used to monitor cardiac output and vascular resistance, these devices are expensive and may be unreliable to apply in patients undergoing liver transplantation, because such patients have hyperdynamic and low-resistance circulation.^[6,7]^ Therefore, despite their invasiveness, thermodilution techniques that use a pulmonary artery catheter continue to be used as routine monitoring devices during liver transplantation.^[8]^ Nevertheless, this technique is limited in not being able to reflect abrupt hemodynamic changes associated with rapid shifts in temperature changes when rushing cold presentative solution into the heart.^[9]^

Photoplethysmography (PPG) waveforms appear as the result of changes in arterial and venous blood, affected by the complex interaction of stroke volume, vascular compliance, and condition of local tissue. Particularly, the amplitude of PPG (PPG_amp_) reflects peripheral blood volume varied according to arterial pulsation, which is well known as the “alternating current (i.e., AC).”^[10]^ Considering that PPG is influenced mainly by pulsatile changes in blood, it can be used to estimate vascular compliance on the pressure–volume loop. Shelley et al suggested that the ratio of changes in PPG to arterial pressure may reflect the effects of vasoconstrictors on vascular compliance, and can be used as a relative compliance measure, regarding PPG_amp_ and arterial pulse pressure (PP) as Δvolume and Δpressure, respectively. They showed that large PPG_amp_ is associated with narrow PP indicating high vascular compliance, and this relationship is reversed after phenylephrine administration.^[11]^ Therefore, we hypothesized that the ratio of changes in PPG to arterial pressure recorded simultaneously would be able to detect the beat-to-beat dynamic changes in vascular compliance associated with postreperfusion syndrome in liver transplantation.

## 2 Methods

### 2.1 Study subjects

We retrospectively evaluated the data of 55 consecutive adult recipients who had undergone living-donor liver transplantation from a prospectively collected registry between January 2015 and May 2015 at our hospital. Among these cases, we selected 26 patients who developed postreperfusion syndrome, defined as a decrease in mean arterial pressure of more than 30% of the value at the end of the anhepatic stage for more than 1 minute within 5 minutes after graft reperfusion.^[2,5,12]^ Five patients were excluded because they had vascular disease including significant obstructive coronary artery disease, carotid artery stenosis, and peripheral vascular disease. Two patients were excluded due to congenital heart disease (atrial septal defect) and pulmonary valve stenosis. None of patients had significant arrhythmia, such as atrial fibrillaion, frequent premature ventricular complex, or complete atrioventricular block. Ultimately, 10 cases were available for analysis after excluding 9 patients with incomplete data on PPG or arterial pressure waveforms, or whose signal quality was poor. We conducted this study after receiving approval from our Institutional Review Board (no. 2016–0571) in accordance with the Helsinki II declaration.

### 2.2 Anesthesia and monitoring

In accordance with standard protocol at our institution,^[13,14]^ we performed routine monitoring using electrocardiography, noninvasive blood pressure monitoring, pulse oximetry, and end-tidal capnography. Anesthesia was maintained with continuous infusions of fentanyl and vecuronium under 1 to 1.5 vol% sevoflurane in a 50% O_2_/air mixture. We monitored direct arterial blood pressure by radial artery catheterization. The pulmonary artery catheterization (7.5 French, Swan-Ganz Ccombo V; Edwards Lifesciences, Irvine, CA) was performed via central venous access (9 French, MAC; Arrow International Inc., Reading, Pennsylvania) and connected to a Vigilance device (Vigilance II; Edwards Lifesciences, Irvine, CA).

### 2.3 Surgical procedure and hemodynamic management

In the anhepatic phase, all patients underwent partial clamping of the inferior vena cava using a piggyback technique without venovenous bypass. For preservation of the liver graft, histidine-tryptophan-ketoglutarate solutions were used in all cases. Before reperfusion of the hepatic graft, the serum levels of potassium and pH were maintained at < 4.5 mmol/L and >7.35, respectively, and the target serum level of calcium was >0.8 mmol/L. Central venous pressure was adjusted to 6 to 8 mm Hg using fluid and blood products. The donor hepatic graft was not flushed with the patient's own blood or colloid solution to remove the preservation solution. After reperfusion of graft, epinephrine of 10 to 20 mcg was used as the first choice of drug and incrementally administered until blood pressure is restored, if the spontaneous recovery of postreperfusion syndrome is delayed.

### 2.4 Data collection and analysis

Electronic medical records of hemodynamic variables of recipients were routinely recorded during the liver transplantation with a computerized data-acquisition system (DI-720U, DATAQ Instruments, Inc., Akron, OH). The waveform variables recorded included continuous electrocardiography, arterial blood pressure, central venous pressure, pulmonary arterial pressure, capnography, and airway pressure, all of which were digitized at a sampling rate of 1000 Hz. In the same way, we also recorded PPG waveforms using a pulse oximetry monitoring device (Cardiocap II, Datex Instrumentarium Corp., Helsinki, Finland), which serves full-scale waveforms without autogain. A pulse oximeter sensor (Nellcor^TM^; Covidien, Boulder, CO) was placed over the index finger on the same side as the radial artery catheterization.

Arterial blood pressure and PPG waveforms were retrieved from the beginning of fall to the nadir in systolic blood pressure immediately after reperfusion, before administration of epinephrine. Beat-to-beat data were extracted using a peak detector (CALC package of Windaq software; DATAQ Instruments, Inc., Akron, OH). We calculated the beat-to-beat ratio between the amplitude of PPG and arterial pulse pressure (hereby denoted as PPG_amp_/PP), which served as relative vascular compliance because of uncalibrated PPG signals.^[11]^ To estimate beat-to-beat cardiac output, stroke volume, total peripheral resistance (TPR), and arterial Windkessel compliance (C_wk_), we analyzed arterial pressure waveforms with Beatscope software (1.1a, Finapres Medical System, Amsterdam, The Netherlands), employing the Modelflow technique.^[15]^ The Modelflow algorithm incorporates age, sex, height, and weight information of the patient to estimate aortic cross-sectional area, and provides beat-to-beat hemodynamic variables.

### 2.5 Statistical analysis

Demographic and hemodynamic data are presented as median with interquartile range (IQR). Appropriate changes in hemodynamic variables between those before and after graft reperfusion were tested using a paired *t* test. For intraindividual data, we performed linear and curvilinear regression between PPG_amp_/PP and TPR or C_wk_, and the best fit was retained. Correlation coefficients were shown as median with 95% confidence intervals (CIs). To analyze data points of all patients, we calculated percent changes from baseline value in PPG_amp_/PP, TPR, and C_wk_. Relationships between percent changes in variables were also examined using linear and curvilinear regression. SPSS software version 21.0 (IBM SPSS Inc., Chicago, IL) and SigmaPlot (version 12.0; Systat Inc., San Jose, CA) were used for statistical analysis.

## 3 Results

**Figure 1:**
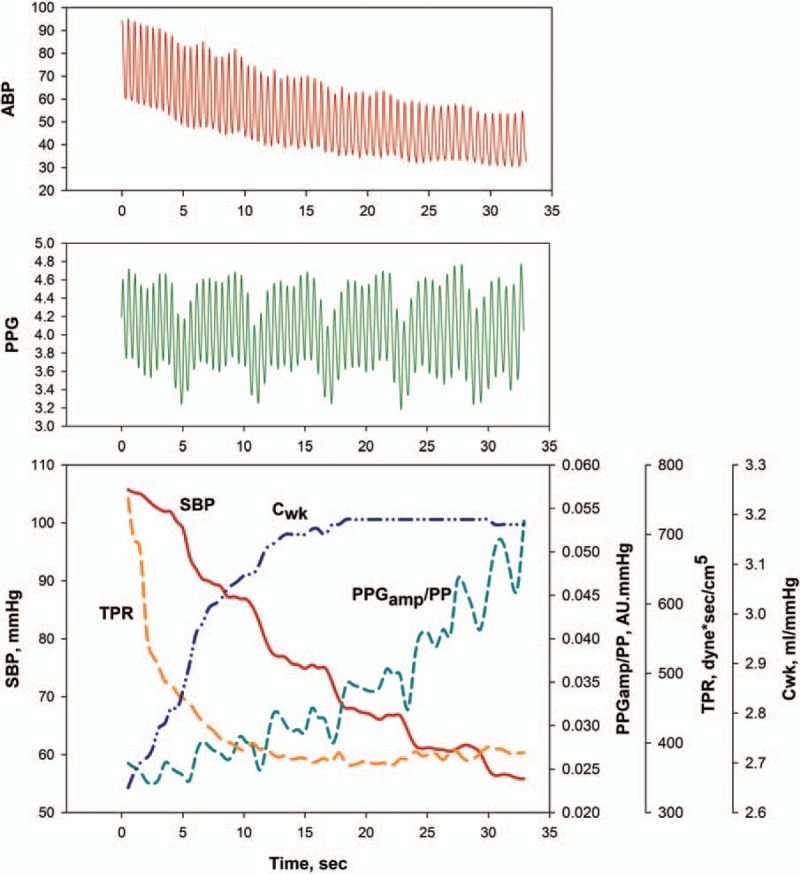
Example tracing of arterial pressure waveforms (upper panel) and photoplethysmography waveforms (middle panel) and example of serial changes in systolic blood pressure, the ratio of the amplitude of plethysmography to arterial pulse pressure, total peripheral resistance, and arterial Windkessel compliance during hypotension after graft reperfusion. C_wk_ = arterial Windkessel compliance, PPG_amp_/PP = the ratio of the amplitude of plethysmography to arterial pulse pressure, SBP = systolic blood pressure, TPR = total peripheral resistance.

Data on 10 patients were available for analysis, and we obtained 517 beat-to-beat data points, which consisted of a median of 50 beat-to-beat points (IQR 42–62) in each patient. Table 1 lists the baseline clinical characteristics of individual patients. Immediately after graft reperfusion, systolic blood pressure was abruptly lowered from 110 mm Hg (IQR 101–126 mm Hg) to 63 mm Hg (IQR 59–67 mm Hg) (–43.6%[IQR–50.3 to –39.3%]). PP was decreased by 24 mmHg (IQR –27 to –14 mm Hg) (–42.1%[IQR–47.8 to –39.0%]), whereas PPG_amp_ after reperfusion was not significantly different compared to that before reperfusion (*P* = .26). Cardiac output increased but to statistically nonsignificant level (*P* = .05), although stroke volume increased from 60 mL (IQR 44–80 mL) to 77 mL (IQR 63–97 mL) (*P* = .004). The PPG_amp_/PP ratio increased by 41.5% (IQR 29.8–88.5%) and C_wk_ increased by 42.0% (IQR 26.4–60.9%). TPR markedly decreased by 46.4% (IQR –56.0 to –33.6%) (Fig. 1 and Table 2).

**Table 1:**
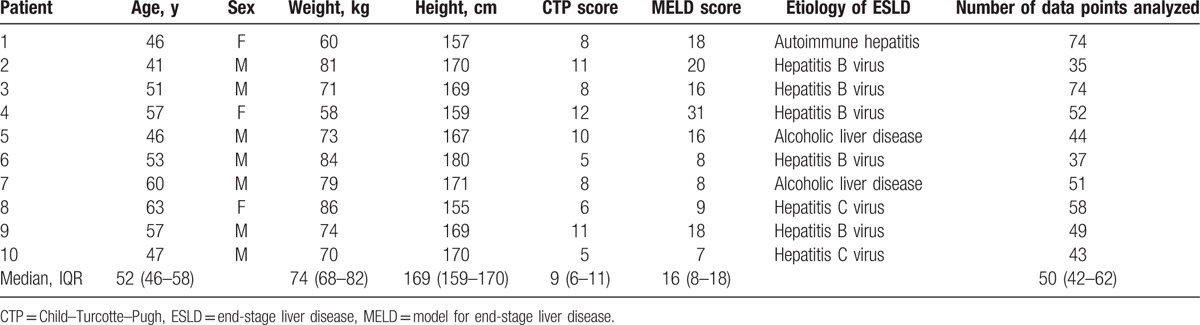
Demographic data in individual patients.

**Table 2:**
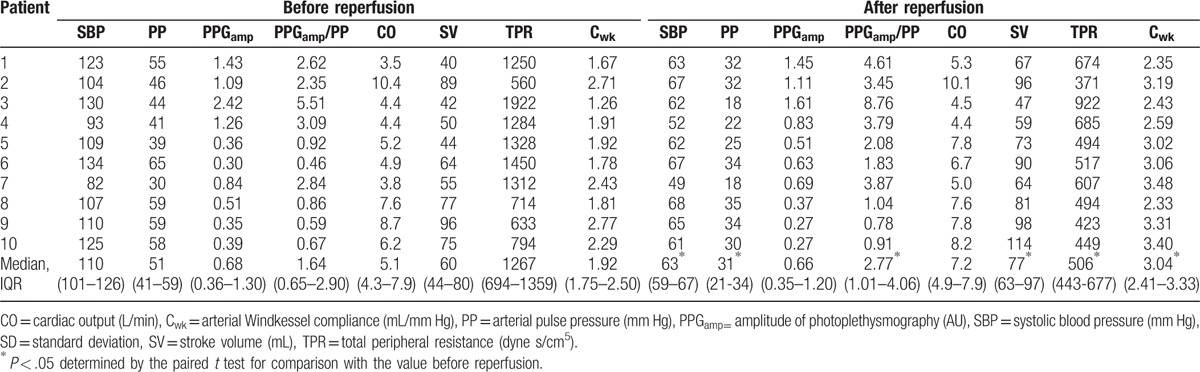
Changes in hemodynamic variables in individual patients.

**Figure 2:**
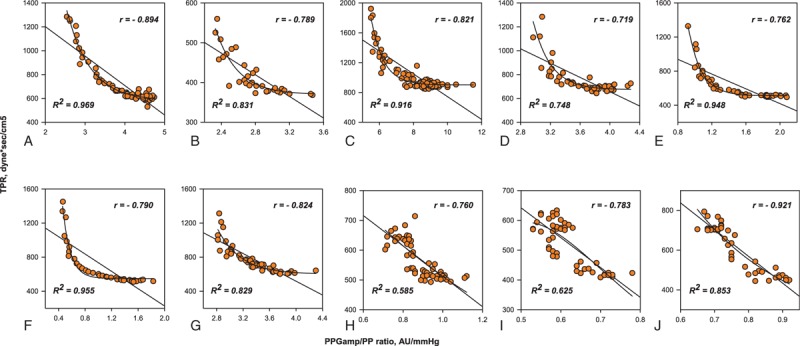
Individual data for the linear and curvilinear relationships between the ratio of the amplitude of plethysmography to arterial pulse pressure and total peripheral resistance. AU = arbitrary unit, PPG_amp_/PP = the ratio of the amplitude of plethysmography to arterial pulse pressure, TPR = total peripheral resistance.

**Figure 3:**
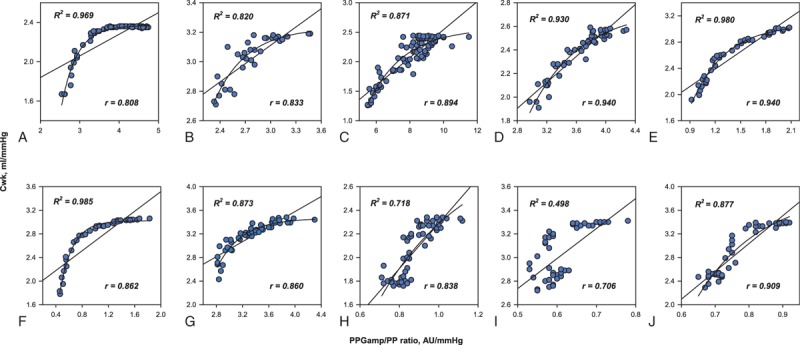
Individual data for the linear and curvilinear relationships between the ratio of the amplitude of plethysmography to arterial pulse pressure and arterial Windkessel compliance. AU = arbitrary unit, C_wk_ = arterial Windkessel compliance, PPG_amp_/PP = the ratio of the amplitude of plethysmography to arterial pulse pressure.

**Figure 4:**
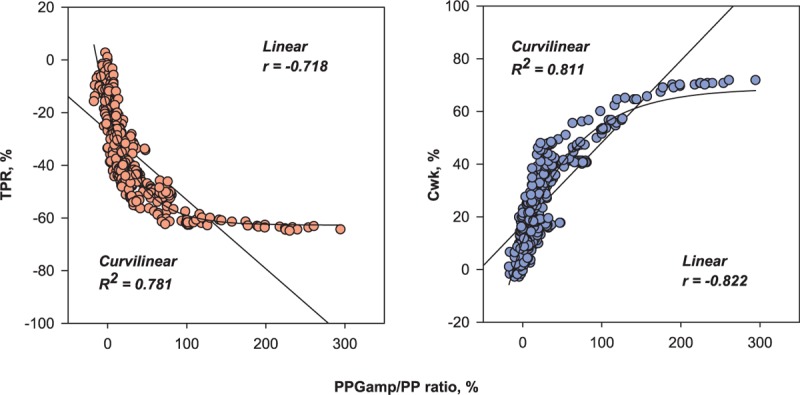
Correlations between percent change in the ratio of the amplitude of plethysmography to arterial pulse pressure and percent change in total peripheral resistance or arterial Windkessel compliance for all data points (517 data points). C_wk_ = arterial Windkessel compliance, PPG_amp_/PP = the ratio of the amplitude of plethysmography to arterial pulse pressure, TPR = total peripheral resistance.

The PPG_amp_/PP ratio was negatively correlated with TPR (median *r* = –0.80 [95% CI –0.85 to –0.76] on linear regression. The association between the PPG_amp_/PP ratio and TPR had a curvilinear relationship and exponential decay curve (median *R*^2^ = 0.84 [95% CI 0.73–0.92]) (Fig. 2). On linear regression, the PPG_amp_/PP ratio was positively correlated with C_wk_ (median *r* = 0.86 [95% CI 0.81–0.91]). On curvilinear regression, it also increased exponentially to the maximum curve (median *R*^2^ = 0.88 [95% CI 0.75–0.96]) (Fig. 3). For all data points, there was a significant correlation between percent change in the PPG_amp_/PP ratio and percent change in TPR (*r* = –0.718 on linear regression and *R*^2^ = 0.781 on curvilinear regression). Percent change in the PPG_amp_/PP ratio of all data points was also strongly associated with percent change in C_wk_ (*r* = 0.822 on linear regression and *R*^2^ = 0.811 on curvilinear regression) (Fig. 4).

## 4 Discussion

Although hemodynamic monitoring has been evolving toward continuous real-time evaluation, it still remains challenging to assess arterial compliance or resistance during the period of graft reperfusion in liver transplantation. In the present study, we estimated relative vascular compliance using a calculation in which the PPG_amp_/PP ratio served as the ratio of changes in volume to changes in pressure. On the basis of a beat-to-beat analysis, we found that the PPG_amp_/PP ratio was strongly correlated with TPR and C_wk_ when hypotension related to postreperfusion syndrome abruptly developed.

The PPG waveform is influenced by pulsatile volume and vascular tone, which can be obtained noninvasively from clinical routine monitoring. Hence, PPG has been investigated to detect arterial compliance, resistance, and stiffness in various studies.^[11,16–18]^ Colquhoun et al^[19]^ showed that the pressure–volume area derived from PPG and arterial waveforms is well correlated with systemic vascular resistance in patients undergoing liver transplantation, but is no better at reflecting vascular resistance than is mean arterial pressure. The reason may be that they measured the average values of pressure–volume area within an identical time block, and compared this index to systemic vascular resistance via pulmonary artery catheter. On the other hand, Shelley et al^[11]^ investigated the possibility of using the PPG_amp_/PP ratio to reflect the response to vasopressor agents in a beat-to-beat fashion. However, they did not investigate the correlation between the PPG_amp_/PP ratio and systemic vascular resistance. Therefore, our study adds value in the sense that we showed the feasibility of using the PPG_amp_/PP ratio for beat-to-beat monitoring of arterial vascular characteristics in clinical situations of abrupt hemodynamic perturbations.

The PPG_amp_ tends to decrease after hypovolemic vasoconstrictive challenge, but the PPG_amp_ of the index finger has been reported to be insensitive to vasomotor changes and to show a low correlation with systemic vascular resistance.^[16]^ Consistent with this finding, we here observed that PPG_amp_ either increased, decreased, or remained unchanged during sudden hypotension. Nevertheless, the PPG_amp_/PP ratio increased in all cases, which may be attributable to the markedly decreased PP (median −42.1%), whereas the change in PPG_amp_ was relatively small. Consequently, these findings support the fact that PPG_amp_ alone is limited when estimating vascular characteristics in peripheral arteries during sudden hemodynamic changes.

In the present study, we also found that relationships between the PPG_amp_/PP ratio and TPR or C_wk_ fitted better to curvilinear regression than linear regression. We speculated that the reason was that estimation of arterial compliance could be affected by the measurement site. The PPG_amp_/PP ratio was obtained from peripheral sites of the finger and radial artery, which can be considered peripheral arterial compliance. Meanwhile, TPR and C_wk_ were determined using the Modelflow algorithm, which estimates aortic flow pulsation by simulating aortic characteristic impedance, arterial compliance, and systemic vascular resistance.^[15]^ Considering the differences between the aorta and peripheral vascular system, a gradual increase in peripheral arterial compliance may be persistent even after fully increased central arterial compliance, resulting in a plateau in a curvilinear curve. Meanwhile, in patient 8, 9 and 10, the relationships between PPG_amp_/PP ratio and TPR or C_wk_ may be shown to fit better linear interpolation. It is speculated that TPR or C_wk_ may persistently increase with the increase of PPG_amp_/PP ratio until the nadir in systolic blood pressure was reached in these patients.

Postreperfusion syndrome can be associated with poorer outcomes after liver transplantation if there is persistent hypotension despite treatment.^[2,12]^ Severe hypotension that develops during the postreperfusion period is associated with lower systemic vascular resistance, higher compliance, and/or reduced cardiac output.^[5]^ Therefore, the identification of vascular characteristics helps guide treatment appropriately. Unfortunately, however, arterial pressure contour analysis can be unreliable under circumstances of alterating vasomotor tone, particularly in patients with advance liver cirrhosis.^[6,7]^ The thermodilution technique using pulmonary artery catheterization also cannot provide continuous cardiac output and systemic vascular resistance under conditions of thermal noise.^[9,20]^ This method takes about 10 to 15 minutes for stabilization.^[20,21]^ In fact, we observed that the Vigilance device could not display hemodynamic parameters even in stat mode. Currently, the use of transesophageal echocardiography (TEE) has been increasing for intraoperative monitoring in liver transplantation, because it is helpful for assessing real-time ventricular contractility and preload. Although it has been proposed that vascular stiffness can be calculated using aortic blood flow and diameter,^[22]^ TEE still has shortcomings and cannot provide continuous information about vascular compliance.^[5,23]^ Therefore, further research is needed to investigate that our simple index together with TEE can be used complementarily to assess hemodynamic status during the reperfusion period.

The present study had some limitations of note. First, we used the Modelflow method to generate beat-to-beat reference values. This method computes cardiac stroke volume by analyzing the pulse wave contour of the arterial blood pressure wave and we used it because we could not directly measure vascular characteristics. The Modelflow method allows for estimation of beat-to-beat stroke volume and derivatives, but its variables have not been calibrated.^[24]^ In addition, as a unit of PPGamp is arbitrarily given, the PPGamp/PP ratio should be regarded as relative compliance.^[11]^ Therefore, we also performed correlation analysis using TPR and C_wk_ data and the PPG_amp_/PP ratio either as absolute values or relative changes. Second, we measured PPG at the index finger, which is markedly sensitive to sympathetic activation.^[25]^ During the anhepatic phase in liver transplantation, sympathetic activation and elevation of systemic vascular resistance are caused by decreased cardiac output related to vascular clamping.^[26]^ Therefore, this effect may influence the calculation of the PPG_amp_/PP ratio even if PPG_amp_ shows insignificant changes during reperfusion.

## 5 Conclusion

Our current results suggest that analysis of the relationship between plethysmographic and arterial pressure waveforms can provide information on beat-to-beat vascular compliance and track the abrupt changes in vascular characteristics associated with hypotension during liver graft reperfusion. This simple index could contribute to differential diagnoses of sudden hypotension and thus could help inform decisions as to which treatments are adequate during this critical period.
